# Dyslexia and Stuttering: An Overview of Processing Deficits and the Relationship Between Them

**DOI:** 10.7759/cureus.47051

**Published:** 2023-10-15

**Authors:** Sami A Algaidi, Amal M Sunyur, Khadija M Alshenqiti

**Affiliations:** 1 Anatomy, Taibah University, Medina, SAU; 2 Medicine and Surgery, Taibah University, Medina, SAU

**Keywords:** learning disabilities, speech disorders, stuttering and dyslexia, dyslexia, stuttering

## Abstract

Stuttering and dyslexia are two processing deficits that have an impact on a person's social and academic lives, especially as they usually affect the pediatric population more than adults. Even though they affect different domains, they have similar characteristics in their pathogenesis, epidemiology, and impact on life. Both disorders represent a considerable percentage of the population worldwide and locally in Saudi Arabia, and they have similar epidemiological trends. Family history, genetic factors, early fetal and neonatal factors, and environmental factors are all identified as risk factors for both conditions. Moreover, it has been established that both diseases share a common genetic and anatomical basis, along with a mutual disruption of diadochokinetic skills. While rehabilitative techniques can be used in both conditions, stuttering could also benefit from pharmacological interventions. This review emphasizes that extensive research should be done to explore both of these conditions as they impact different areas of one's life and the relationship between them to better understand their pathophysiological origins.

## Introduction and background

Speech and language disorders are classified into many different categories, including stuttering, speech-sound disorder (SSD), specific language impairment (SLI), and developmental dyslexia (DD) [1].

The term "dyslexia" is drawn from the Greek words dys (deficiency or lack of) and lexicon (word). Dyslexia occurs when an individual has significant difficulties reading, writing, and understanding a text. Unlike other learning disabilities, intelligence is not affected, so people with dyslexia have adequate intelligence and typical schooling, and it is not essentially an all-or-nothing condition; the patient can exhibit a different degree of severity [2,3].

Stuttering is a speech disorder characterized by involuntary, audible, or silent repetitions or prolongations of sounds or syllables, along with disturbances in the fluency of verbal phrases. It is not easily controllable and may co-occur with other movements as well as negative emotions like fear, embarrassment, or agitation, and it is associated with higher levels of social anxiety [4,5]. Being attentive is important for making a correct diagnosis in children, as it is believed that early therapy is critical. Stuttering in adults could be associated with significant psychosocial morbidities, such as social anxiety and poor quality of life [6].

As much as the two sound very different, it has been noted that the two disorders share commonalities in their anatomical basis, pathogenesis, and epidemiological features. Both of which were found to have an underlying difficulty in processing speech sounds in a word, which is why they are collectively termed processing deficits [7].

According to the review of literature we made, there are just a few studies that have discussed dyslexia and stuttering and whether or not a relationship between them exists. So, our review article's main aim is to provide an overview of the global and local epidemiological features of stuttering and dyslexia, along with their risk factors, clinical characteristics, and available methods of management, and explore the relationship between them. We searched for articles to be reviewed in Google Scholar and PubMed. The most recent articles that were published on a certain topic with the best study design (according to the hierarchy of evidence) were included in this study. Therefore, articles with topics that were explored in more recent studies or with a more reliable study design were excluded.

## Review

Local and global epidemiological trends of stuttering and dyslexia 

Stuttering is not an uncommon problem in children. The prevalence of stuttering varies significantly; however, it has been estimated to be around 0.72% of the general population [8]. Approximately 5% of all children experience a six-month or longer period of stuttering. Later in childhood, about 75% of kids with stuttering will start to recover, and only 1% of them will have long-term consequences [9].

Locally in Saudi Arabia, a study about the social knowledge of stuttering in the Saudi population discussed the prevalence of stuttering in KSA. According to the findings, stuttering has a higher incidence at young ages, and IQ scores do not correlate with the prevalence of stuttering in the Saudi community. Furthermore, stuttering affects more than 6% of the Saudi population, and it is dramatically increased in males compared to females. Information about the effects of stuttering, on the other hand, was rarely reported. As a result, future experiments with well-planned public education and health interventions for stuttering will be encouraged [6].

Regarding the age of onset of stuttering, it is known to predominantly affect children, and different studies have explored the age of onset and the people at risk of developing stuttering. Studies showed that most stuttering cases start in early childhood, sometimes even before 18 months of age; only a few cases reported stuttering that started during the teens [10]. In a study about the onset of stuttering in children, most children in the study were noticed by their parents to have begun stuttering for periods of two months or less. A study shows data from six studies in the United Kingdom, Denmark, the USA, and Australia about the onset of stuttering among school and preschool children (till the age of six). Approximately 60% of onsets are between 24 and 35 months of age; 95% of onsets happen at 48 months of age; and only 5% of cases have begun stuttering over the age of four. Conversely, in Denmark's schools, results show that 95% of stuttering onset is over the age of four. There is a remarkable difference between studies on the age of onset and the percentage of children. Moreover, about the patterns of stuttering, most parents said that their children's syllable and word repetitions (especially short ones) were common in early stuttering, and syllables were repeated three to five times. Sound prolongations, silent intervals, blocks in short-syllable and single-syllable word repetitions were seen in a lower percentage of children, which are all symptoms of children who stutter [8].

As for dyslexia, it is one of the most common learning disorders and the most common cause of difficulties with reading, writing, and spelling. The global prevalence of dyslexia is estimated to be between 5% and 10% of the population, but it may be as high as 17% [11].

Among the Saudi population, a cross-sectional study conducted in Riyadh, Saudi Arabia, in 2015 at public and private girls' schools, which included a random sample of 720 students from 1st grade to 6th grade, showed that 172 out of 720 students (23.89%) have some sort of learning difficulty. The most common of all types of learning disabilities were dyslexia and dysgraphia, which represent 31.4% and 27.3%, respectively, as shown in Table 1. Academic performance is lower for students with learning difficulties than for normal students. The results showed dyslexia was the most common learning difficulty among 720 students, with a percentage of 31.4%, which represents 7.5% of the Saudi population [7].

**Table 1:** Results of the learning disability study among the Saudi population. This table was adapted from [7].

Learning Disability	Frequency	Percentage
Dyslexia	54	31.4%
Dysgraphia	47	27.3%
Dyscalculia	19	11.1%
Others	52	30.2%
Total	172	100%

Risk factors of stuttering and dyslexia

In the case of stuttering, the presence of monosyllabic word repetitions and sound prolongation is related to emotional stress, according to an analytical cross-sectional study that was conducted at the Fluency Studies Laboratory of a public university's Department of Speech and Hearing Disorders. Also, having relatives with stuttering, a history of delayed childhood development, and a late start to speech or learning were mentioned as factors that increase the risk of speech disorders. Physical stress and inappropriate family attitudes were also implicated in the development of stuttering [12,13]. A secondary analysis of data done in 2010 identified being fidgety and restless as a risk factor, which might point towards the coexistence of attention deficit hyperactivity disorder (ADHD). Also, this study found that troubles during birth and parental alcohol abuse are possible factors that may cause stuttering [14]. Family history of stuttering, unfavorable performance in phonological assessment, more frequent stuttering-like dysfluencies, and poorer nonword repetition task performance were linked as risk factors for the persistence of childhood stuttering rather than its development [15].

As for dyslexia risk factors, they may include premature birth, prenatal alcohol consumption, and nicotine exposure, which may alter brain development in the fetus [16]. In 2012, a study identified maternal smoking during pregnancy, birth weight, and socioeconomic status as possible environmental risk factors for developmental dyslexia, as they are thought to increase the genetic liability to developing dyslexia [17]. Moreover, a study by the same research group in 2013 identified the risk of miscarriage, maternal and paternal age at childbirth, and the educational level of both parents during the first three years of the child as potential risk factors [18]. Also, a cross-sectional study investigating the prevalence and risk factors of dyslexia in China found that a child’s degree of engagement in active learning was proposed to be a possible factor contributing to the development of dyslexia [19]. The frequency of literacy-related activities, time spent on electronic devices, and restrictions applied to children on the use of electronic devices have also been implicated in the development of this condition [20].

As for the genetic risk factors, a systematic review by Becker et al., 2017, found that many genes were discussed for their possible association with the development of dyslexia (i.e., DYX1C1, DCDC2, DYX9, and DYX2). However, no evidence that the proposed genes play a role was investigated in another study [21]. Despite that, a study conducted by the University of York's Psychology Department Ethics Committee and the NHS Research Ethics Committee found that having a family history of dyslexia or other learning disorders is one of the main risk factors. Dyslexia has a hereditary aspect as it runs in families; this has been a known fact for many years, and there is a lot of evidence of its association with candidate genes. However, these hereditary (familial) effects are not very clear because learning disabilities are influenced by both genetic and environmental factors. Even if other risk factors were controlled, people with dyslexia would still not be able to learn like normal people, which is explained by gene-environment correlation. Because a parent's genotype is related to both the child's genotype (here, the child has a genetic basis of dyslexia) and the child's environment (for example, poor quality education), it represents a passive gene-environment relation (rGE), while active rGE refers to children actively selecting their environment for genetically influenced reasons, which means that the child has genetic bases but also does not want to study [22].

Clinical presentation of stuttering and dyslexia

Stuttering is a speech disorder in which the flow of speech is disrupted by repetitions or prolongations of words, syllables, and sounds. Patients with stuttering might have difficulties starting words, too. Short pauses or silences between syllables or sentences or within a word (broken word) and excessive tenseness, tightness, or movement of the face or upper body to pronounce a word are also features of the disorder. Moreover, it may be accompanied by excessive eye blinking and jaw or lip tremors. Symptoms of stuttering tend to be exacerbated by public speaking or anxiety; they are often better when singing or speaking alone [13].

Dyslexia symptoms are often hard to detect before school when the child is noted to have learning difficulties. Symptoms before school often include late speech, slow learning of new sentences and phrases, issues with forming sentences, and difficulties with memorizing names, colors, or letters. Once the child starts school, signs and symptoms become more obvious and easier to notice, which may include his or her learning level being below the normal level of his age, difficulty recognizing (and hearing) similarities and variations in letters and words, difficulty with pronouncing new and unfamiliar words, spelling issues, and spending a longer time on an assignment that requires reading or writing. In teens and adults, dyslexia may manifest as effortful and sluggish reading and writing, avoiding reading and writing activities, issues with the pronunciation of names or words, problems retrieving words, difficulty learning a new language, and problems with memorization [16].

Pathogenesis of stuttering and dyslexia

Is it possible that stuttering-related candidate genes play a role in dyslexia pathogenesis? Mutations were discovered in the GNPTAB, GNPTG, and NAGPA genes in a study that began the analysis of candidate genes [23]. In another stuttering study, researchers found a mutation in the GNPTAB gene, as well as the other two related genes, GNPTG and NAGPA, in large families and sporadic patients, confirming their relationship with stuttering [24-26]. Children with motor deficiencies or learning disabilities are more likely to stutter, suggesting that speech disorders and learning disorders are genetically related. As a result, these three genes (GNPTAB, GNPTG, and NAGPA) could predispose people to stuttering and may also be a risk factor for other learning and speech disorders. The genetic architecture, which includes a variety of molecular mechanisms, is complex. Rare protein-coding mutations in the forkhead box P2 (FOXP2) transcription factor, for example, cause serious defects with speech sound sequences, while common small-effect genetic risk variants in genes like CNTNAP2, ATP2C2 and CMIP are related to typical types of language impairment [27].

Using functional neuroimaging techniques, it has been suggested that dyslexia originates from abnormal activation of language networks of the left hemisphere, particularly the temporoparietal region, which is believed to be important for phonological processing, and the occipitotemporal region, which is important for visual word recognition. It has been reported that a decrease in gray matter may exist in these regions [28].

Stuttering was once thought to be mainly a psychogenic condition, and the severity of stuttering is affected by arousal, nervousness, and other factors. As a result, two-factor models of stuttering have emerged. The first factor, which is most likely a structural or functional central nervous system (CNS) abnormality, is thought to be the cause of the condition. The second factor is the avoidance of learning, which strengthens the first one [29]. Differences in brain anatomy, function, and the regulation of dopamine levels have been associated with stuttering, which is thought to be attributed to genetic factors [6]. On the other hand, it is also hypothesized that stuttering is a result of genetic, environmental, and epigenetic interactions that shape the child’s anatomy and function of the brain. It was noticed that stuttering results from defective interarticular coordination patterns, which are well-functioning in fluent adults. This is hypothetically attributed to the inability of the central nervous system to develop well-functioning muscle synergy and coordinated motor programs. These unstable patterns are suggested to originate from abnormal left speech motor and premotor areas and abnormal connections between motor, language, and auditory areas. Recently, a delay in the development of speech-motor control is thought to play a role in early dyslexia, which contributes to its persistence [30].

Management of stuttering and dyslexia 

Stuttering can be treated in different ways, according to a review by Maguire et al., 2020. First, pharmacological therapy includes first-generation dopamine-blocking medications and a variety of second-generation dopamine-blocking medications; the first generation has more serious side effects than the second generation. Even though numerous studies have demonstrated the efficacy of other pharmacological treatments in reducing disease burden, such as alpha receptor agonists, GABA agonists, and calcium channel blockers, no drugs have been approved by the FDA until now. Recently, there have been two active drugs, ecopipam and valbenazine, and they are currently undergoing clinical trials. The second option is speech therapy, which has shown improvement in patients with stuttering. Finally, cognitive behavioral therapy is a psychotherapeutic method that can show better outcomes for patients with stuttering; it is also associated with reducing anxiety, and the patients will be more confident about speaking in public. According to a clinical study of cognitive and behavioral therapy (CBT) combined with speech therapy, stuttering is similar to other neuropsychiatric disorders like depression, in which "talk" therapy combined with medication is the best treatment option. Moreover, CBT is useful in cases of stuttering, as it often coexists with social anxiety and other anxiety disorders. Because of their experience with both psychotherapy and psychotropics, psychiatrists, along with phoniatric doctors, speech-language pathologists, and psychologists, play a significant role in the medical team to help improve stuttering outcomes. Psychiatrists should collaborate with these clinicians. Transcranial direct current stimulation is also under investigation for its ability to improve disfluency, as some evidence of its efficacy in improving fluency is available [31].

The Lidcombe program (LP) is also used for the treatment of stuttering, and it has shown promising evidence. LP is a method by which the parents of a child with stuttering are instructed to verbally describe a situation to the child and ask him to say a sentence that is similar in both length and complexity. LP is considered a direct method of treatment as it engages the child in an activity that improves his speech. The Lidcombe program was shown to improve speech fluency in children who are younger than six years of age. Several other methods have shown promising results in improving stuttering, such as the RESTART-DCM program, which indirectly aims to improve stuttering by balancing the communication demand and the child’s ability, and the parent-child interaction (PCI) program, which aims to improve stuttering through interactive strategies. In adolescents and adults, speech restructuring therapy, which is often used in conjunction with transcranial direct current stimulation, has been shown to be effective in reducing stuttering and improving fluency, but with no effect on other aspects of stuttering like social anxiety [32].

Technology-based therapy is an emerging technique in the management of stuttering. It includes virtual reality-based interventions, video-self modeling, telehealth technology, biofeedback, software programs, and other forms. Technology-based stuttering interventions have been shown to be well-tolerated and effective in reducing stuttering, according to a systematic review [33].

As for dyslexia, several treatment options exist, including programs for reading where children with dyslexia who have trouble matching letters to sounds and words to meanings are given additional reading and writing assistance. The kids will work with a reading teacher; they will learn how to pronounce letters and words (phonics), read more quickly, and comprehend what they're saying.

A few reading programs are designed specifically for dyslexic children. For instance, Orton-Gillingham is a step-by-step method for teaching children how to match letters to sounds and identify letters in words. Multisensory instruction teaches children how to learn new skills using all of their senses (touch, sight, sound, smell, and movement). To learn how to read different words, the children may run their fingers over sandpaper letters. Individualized for children with learning disabilities such as dyslexia, an individualized educational program (IEP) outlines the child's requirements and how the school can assist with meeting those requirements. Every year, the plan will be updated by the parents and the school based on the child's improvement. A learning specialist may do one-on-one or group sessions, either in the classroom or in a different room in the school, to provide special education to the child. Accommodations are outlined in an IEP, which describes special programs for a child's needs to make learning easier. For instance, audiobooks provide additional time to complete tests or text-to-speech, which is a technology that reads words out loud from a machine or document. A child's education should be a continuous process that is not limited to the classroom. Reading with the children and assisting them in sounding out words that they are having difficulty with is a good idea.

Moreover, some suggestions were shown to help children and adults with dyslexia, like trying to read in a quiet environment, listening to books on CD or device and reading along with the narration, asking teachers and colleagues for assistance when needed, joining a dyslexia support group for kids or adults, and getting enough sleep along with eating a well-balanced diet [34].

Furthermore, technology-based interventions have also been shown to improve phonological skills in dyslexic children, according to a meta-analysis of four studies [35].

Relationship between dyslexia and stuttering 

In terms of epidemiological characteristics, the prevalence of dyslexic adults who had a speech disorder (stuttering) in childhood was indicated in the study with a percentage of 34%. This was determined by the severity of dyslexia. People with moderate dyslexia had a lower incidence of childhood stuttering (15%), while those with severe dyslexia had a higher incidence of childhood stuttering (47%). Furthermore, the prevalence of adults with stuttering who had dyslexia as children was 50%. Phonological working memory, perception, and retrieval were equally decreased in adults with dyslexia and adults with stuttering. According to the finding, stuttering and dyslexia may share a phonological deficit [10]. Moreover, both diseases have a large male bias (2:1 ratio for dyslexia and 3.7:1 ratio for stuttering) [36].

Regarding the pathogenesis of both conditions, several studies have suggested that speech and language disorders may share a genetic basis. For example, the forkhead box P2 (FOXP2) gene has a significant role in the pathogenesis of stuttering and dyslexia [1,37,38]. Although stuttering and dyslexia seem to be quite different diseases, they have some significant similarities. Both disorders have been shown to share the same genetic factors (i.e., DRD2, GNPTAB, and NAGPTA) [24,39]. In both disorders, phonological processing is impaired (e.g., phonological awareness [40] and phonological working memory [41].

Furthermore, one study discussed the common pathogenesis from a different angle, which is the diadochokinetic skill. The sample of the study includes dyslexic, stuttering, and normal children. The amount of time needed to receive and interpret motor gestures (which are usually short, repetitive, rhythmic movements that are closely tied with prosody in verbal speech) to produce precise and repeated syllables over time is known as diadochokinetic skill. Verbal diadochokinetic skill is the time required for oral recurrence of monosyllable and multi-syllable verbal structures, which is usually measured using the maximum recurrence rate paradigm.

Most studies indicate that when a child's motor system matures, their diadochokinetic skills improve, and by the age of 9-15, they should be comparable to an adult's motor system. The study we found started with 120 children: 40 with stuttering, 40 with dyslexia, and 40 who were normal. Age, gender, and bilingualism were evenly distributed among the three groups. The inclusion criteria were children from 6-11 years old with a diagnosis of stuttering for the stuttering group and dyslexia for the dyslexic group. The diadochokinetic skills of the selected children were assessed separately using a diadochokinetic task to measure their diadochokinetic abilities. Children were asked to repeat the monosyllables (pa/ta/and/ka), which were presented one by one orally in a quick, accurate, and fluent way. The examiner used a chronometer (an instrument for measuring time accurately despite motion or variations in temperature, humidity, and air pressure) to measure the time that was needed for every child to do 15 repetitions. To assess the ability of children for long syllable (pataka) pronunciation, the same method was used, and the time spent on 15 repetitions was reported. The control group underwent a similar procedure. Finally, the study's findings show that children with stuttering and children with dyslexia have inefficient movement of the tongue, particularly in terms of diadochokinetic skills. Deficits indicate that the children's motor and speech functions were affected. Because of the similarity between the two groups, the study suggests that both disorders originate from the tongue. Furthermore, the motor deficits of both disorders can be explained by their shared neural basis. It could also be explained by the fact that in both disorders, the malfunction occurs in a similar region of the brain. To identify this dysfunction, different parts of the children's brains were examined with fMRI while they performed diadochokinetic tasks [42].

## Conclusions

We concluded that stuttering and dyslexia originate from a complex interplay between a genetic predisposition, anatomical abnormalities, and defective neurophysiological mechanisms. Moreover, stuttering and dyslexia seemingly share a relationship in different aspects. Both disorders have been shown to have a similar genetic basis, common anatomical involvement, and comparable pathophysiological processes and neural ground. Also, dyslexia and stuttering have similar epidemiological characteristics. So, it is important to do further research to determine the presence of a relationship between dyslexia and stuttering, which may become helpful for treatment and prevention methods in the future. Furthermore, interventional modalities, such as technology-based interventions, have promising evidence for their potential benefits in managing both dyslexia and stuttering. While speech therapy and cognitive and behavioral therapy remain the most commonly used methods to manage stuttering, other interventional programs like the Lidcombe program, the RESTART-DCM program, and the parent-child interaction program have shown encouraging primary results in the management of stuttering. However, data on all existing interventions need further exploration to ascertain their value in the management of both conditions, as sufficient evidence is not established yet.
